# Strengthening and promoting digital health practice: results from a Global Digital Health Partnership’s survey

**DOI:** 10.3389/fpubh.2023.1147210

**Published:** 2023-06-19

**Authors:** Fidelia Cascini, Andrea Gentili, Francesco Andrea Causio, Gerardo Altamura, Andriy Melnyk, Flavia Beccia, Ciro Pappalardo, Alberto Lontano, Walter Ricciardi

**Affiliations:** Section of Hygiene, University Department of Life Sciences and Public Health, Università Cattolica del Sacro Cuore, Rome, Italy

**Keywords:** health systems, digital health, governance, communication, promotion, telehealth, electronic health

## Abstract

**Background and objective:**

The capacity to promote and disseminate the best evidence-based practices in terms of digital health innovations and technologies represents an important goal for countries and governments. To support the digital health maturity across countries the Global Digital Health Partnership (GDHP) was established in 2019. The mission of the GDHP is to facilitate global collaboration and knowledge-sharing in the design of digital health services, through the administration of surveys and white papers.

**Objective:**

The scope of this study is to critically analyze and discuss results from the Evidence and Evaluation GDHP Work Stream’s survey, understand how governments and countries intend to address main obstacles to the digital health implementation, identify their strategies for a communication of effective digital health services, and promote the sharing of international based best practices on digital health.

**Methods:**

This survey followed a cross-sectional study approach. A multiple-choice questionnaire was designed to gather data. Choices were extracted from research publications retrieved through a rapid review.

**Results:**

Out of 29 countries receiving the survey, 10 returned it. On a scale from 1 to 5, eHealth systems/platforms (mean = 3.56) were indicated as the most important tool for centralized infrastructure to collect information on digital health, while primary care (mean = 4.0) represented the most voted item for healthcare services to collect information on digital health. Seven Countries out of 10 identified lack of organization, skepticism of clinicians, and accessibility of the population as a barriers to adopt digital health implementation, resulting to be the most voted items. Finally, the most endorsed priorities in digital health for Countries were the adoption of data-driven approaches (6 Countries), and telehealth (5 Countries).

**Conclusion:**

This survey highlighted the main tools and obstacles for countries to promote the implementation of evidence-based digital health innovations. Identifying strategies that would communicate the value of health care information technology to healthcare professionals are particularly imperative. Effective communication programs for clinicians and the general population in addition to improved digital health literacy (both for clinicians and citizens) will be the key for the real implementation of future digital health technologies.

## Introduction

The Fourth Industrial Revolution and the transition from mechanical and analog to digital technology, has changed “the way humans create, exchange, and distribute value” (1).

The Digital Health concept was first introduced in 2000 by Seth Frank (2) and today encompasses fields such as wearable technology, telehealth and telemedicine, customized medicine, mobile health (mHealth), health information technology (IT), artificial intelligence (AI), Internet of things, virtual care, remote monitoring, big data analytics, blockchain platforms, tools enabling remote data capture, storage, and the exchange of data and sharing of relevant information across the health ecosystem (3).

In the healthcare sector, the introduction of digital health has created opportunities at the global level. The World Health Organization has addressed the topic with the release of a “Global strategy on digital health 2020–2025” that aims to “enhance health outcomes by improving medical diagnosis, data-based treatment decisions, digital therapeutics, clinical trials, self-management of care and person-centered care as well as creating more evidence-based knowledge, skills and competence for professionals to support health care” through the implementation of information and communication technology tools worldwide (4).

In recent years, the effects of those disruptive technologies have been transversally investigated on several aspects. The impact of electronic tools (such as patient portals, mHealth, wearable devices, and telemedicine) on health outcomes and system efficiency has showed a generally favorable effect (5–7). Literature regarding the cost-effectiveness of digital health innovations is still limited, but it showed to be cost-effective, especially when it concerns a new mobile application or a web portal intervention (8, 9). Artificial intelligence and machine learning have been used to identify healthcare pathways to citizens and patients, reporting to improve the safety and quality of patient care, enabling the evaluation of clinical risks at each step of the patient journey (10).

With the aim to support the effective implementation of digital health, exchange global best practices, and to provide opportunities for policy co-production and knowledge transfer, the global summit for digital health, named the Global Digital Health Partnership (GDHP), was internationally launched in 2018 (11). It is a collaboration of 33 countries and territories, the World Health Organization (WHO), the Organization for Economic Co-operation and Development (OECD), and the International Digital Health & AI Research Collaborative (IDAIR) (12). Through this collaboration, the GDHP hopes to improve the safety, quality, and effectiveness of healthcare, to support earlier diagnosis of disease, and to promote the development of new medicines and treatments, through the use of digital health services (13).

One of the workstreams within the GDHP is the Evidence and Evaluation Workstream and it aims to share methods of digital health evaluation frameworks and concrete examples of lessons learned from digital health benefits evaluations and strategies. It has already published several whitepapers:

- In February 2019, GDHP released “Measuring Benefits,” an international overview of approaches for evaluating digital health technologies and services (14).
- In July 2020, GDHP released “Benefits Realization: sharing insights,” with the aim to identify a common framework for the evaluation of digital health services and technologies among different countries (15).
- In December 2022, GDHP released “Practicing the evidence,” an international survey aiming to understand which evidence-based practice in digital health is effectively implemented in various countries and regions of the partnership.

During the COVID-19 pandemic, digital health innovations and technologies have emerged worldwide as indispensable resources. They work to guarantee the continuity of care of patients, especially among frail patients with multiple chronic diseases, to improve the surveillance and the control of patients and of communicable diseases and to enhance the adherence to therapy of older adult people (16, 17).

This paper addresses the results derived from the answers of 10 different member countries of the GDHP to our survey. The main tools used by governments and countries to disseminate and communicate effective results of digital health services have been determined. Special attention was also given to the identification of the main barriers to the implementation of digital health services, and to the identification of the top three priority areas to be addressed by countries in the future. A top-down method by directly collecting representative bodies’ answers has been followed (18).

The scope of this paper is to critically analyze how governments and countries intend to address main obstacles to the digital health implementation, identify their strategies for productive communication on the topic of digital health services, report similarities and differences, and promote the sharing of international best practices for the dissemination of effective digital health technologies and innovations.

## Methods

### Rapid review of literature

A rapid review of literature was performed between March and April 2021 in order to identify studies addressing the evaluation of digital health technologies and innovations, to methodologically measure the use and acceptability of digital health interventions.

### Search strategy and data sources

PubMed, Web of Science, and Scopus were the academic databases and search engines used, while “Digital health,” “health digitalisation,” “national plan,” “WHO framework,” “The digital competence framework,” “questionnaire,” “survey,” and “acceptability” are a few of the terms that were included in the search string and linked by Boolean operators. To identify relevant missing records, grey literature research was then carried out utilizing both Google search tool and Google Scholar.

A questionnaire was produced to be presented to the representative of each GDHP nation.

### Inclusion and exclusion criteria

Only original, English-language studies that have accessible full texts, were included. Publications that contained a questionnaire, were published in a peer-reviewed scientific journal and on the evaluation, the usage and the acceptance of digital health services and technology as well as the digital transition of health, were selected.

### Study selection and data extraction

Articles were screened by title and abstract, then by full-text analysis. A spreadsheet on Microsoft Excel^®^ for Windows was used to extract the data. The team established a standard spreadsheet with preset fields to unify the data extraction procedure. From the original studies, several qualitative and quantitative data were gathered. The first author’s name, the year, the kind of questionnaire, the nation, and the digital field were all recorded as qualitative data. Quantitative information was retrieved on the number of recorded responses, reader compliance, evaluated fields, and survey duration.

### Survey development

To collect data, a structured multiple-choice survey/questionnaire was prepared. The research papers acquired during the rapid review were used to extrapolate the survey options. Respondents were encouraged to provide comments in addition to completing the structured questions. Government agencies were regarded as the major stakeholders in this GDHP survey. Supplementary Table S1 contains a list of the participating respondents. Countries, organizations, and territories who are members of the GDHP, were invited to reply to the survey. Only one answer was permitted per question. Six main categories were covered by the survey’s questions: “Priorities identification,” “Relationship between national health plans and criteria for funds allocation,” “Evidence about the development of digital health services,” “Providing digital health evidence,” “Collecting data,” and “Strengthening and promoting digital health.” This article focuses on the category of “Strengthening and promoting digital health.”

### Strengthening and promoting digital health

This section of the research enquires about countries’ communication strategies. It examines the relevant engagement and recruiting approaches for digital health interventions (DHIs), highlighting the current state of communication in each nation and the challenges they face. The 40 different items in this section were divided into five distinct questions (see Table 1), described as follows:

- Question 1: What kind of sources and tools are mainly used in your country to collect information on digital health? (15 items to be evaluated on a scale from 1 to 5).
- Question 2: What has been done at a national level to develop a consolidated communication of digital health technologies? (9 items to be answered with yes or no).
- Question 3: What are the tools your government/country uses for the communication/dissemination of results of digital health services? (7 items to be evaluated on a scale from 1 to 5).
- Question 4: Please indicate if, in your experience, any of the following are barriers to implement digital health use: (9 items to be answered with yes or no).
- Question 5: Which are the three priorities areas that you would like to see future digital health directed towards? (To be answered with an open answer).

**Table 1:** Topics, identification codes, and items of the different questions.

Topic	Identification codes	Items
Tools to collect digital health information	A1	National eHealth system/platform
	A2	National digital health agency
	A3	Institutions/Organizations for digital health
	A4	National digital networking system
	A5	Ambulance monitoring systems
	A6	Ambulance monitoring systems
	A7	Smart hospitals and providers
	A8	Local healthcare monitoring systems
	A9	Online bookings for healthcare services
	A10	Online payments for healthcare services
	A11	Primary care
	A12	Electronic patient web portals
	A13	Mobile-health applications
	A14	Pharmacies
	A15	Drugs monitoring systems
Actions taken to consolidate digital health communication	B1	National laws promoting the digitalization of health at the population level
	B2	Information and advertising campaigns on the importance of digital health
	B3	Communication campaigns on the usefulness, usability and availability of digital health
	B4	Training and engagement of healthcare professionals
	B5	Professional courses promoted by universities
	B6	Advanced university courses
	B7	Programs for schools on digital health
	B8	Increasing the awareness of the population through media and press
	B9	Patient engagement
Tools for communication/dissemination of results	C1	Media
	C2	Scientific publication
	C3	Institutional websites
	C4	Newspaper articles
	C5	Social media
	C6	Video interviews of institutional representatives
	C7	Other
Barriers to implement digital health use	D1	Lack of infrastructure
	D2	Lack of technological equipment
	D3	Lack of political will
	D4	Lack of economic resources
	D5	Lack of organization
	D6	Skepticism of clinicians
	D7	Limited skills of the population
	D8	Accessibility of the population
	D9	Other

### Statistical analysis

A descriptive analysis was performed on the items resulting from the selected questions. Data analysis was performed using the STATA 16^®^ software. For each question and each country, we calculated the mean score as a synthetic measure to compare the different items and countries. In addition, overall scores were calculated for each single item and country. To assess similarities and differences between countries, a dendrogram was created using the STATA^®^ software. Furthermore, Cronbach’s alpha was evaluated. Significant reliability values for Cronbach’s alpha are to be considered those >0.70.

## Results: GDHP promotion and communication of digital health

Out of the 29 countries who received the survey, 10 returned it: Australia, Brazil, Canada, Hong Kong, India, Italy, Netherlands, Poland, South Korea, and the United States. Most countries belong to Europe (*n* = 3) and Asia (*n* = 3), followed by North America (*n* = 2), South America (*n* = 1), and Oceania (*n* = 1).

The number of items from closed-ended questions is 40. These are divided as follows:

- The first group of questions included 22 items to be evaluated on a scale from 1 to 5, divided into three questions (*What kinds of sources and tools are mainly used in your country to collect information on digital health?*)
- *What are the tools your government/country uses for the communication/dissemination of results of digital health services?*
- The second group of questions included 18 items to assess the presence/absence of actions and barriers to implementing digital health (*What has been done at a national level to develop a consolidated communication of digital health technologies? – Please indicate if, in your experience, any of the following are barriers to implementing digital health use*). The total number of quantitatively analyzed elements is 555, resulting from 387 elements in section 1 (three answers missing from Netherlands) and 168 in section 2 (two answers missing from Netherlands).
- In the third section, open questions were asked. The data processed in this section refer to four proposed items: one 5-point Likert question, two multiple-choice questions, and one open-ended question. In addition, a 5-point Likert question on sources and tools mainly used to collect information on digital health (in the section “Providing digital health evidence”) was considered.

The first group of questions: questions to be evaluated on a scale from 1 to 5.

### Tools to collect digital health information

When asked about the role of centralized infrastructures in their country in collecting information on digital health (*What kinds of sources and tools are mainly used in your country to collect information on digital health? Centralized infrastructure*), respondents indicated national eHealth systems/platforms (mean = 3.56) as the most used on average, followed by institutions/organizations for digital health (mean = 3.44), national digital health agencies (mean = 3.33) and national digital networking systems (mean = 2.2).

India scored the highest (mean = 5), whereas the US scored the lowest (mean = 1). This is reflected by the overall score the individual countries achieved on item 1, where India leads (*n* = 20), followed by South Korea (*n* = 17), Hong Kong (*n* = 16), and Canada (*n* = 14). The countries performing the worst are the US (*n* = 4), followed by Italy (*n* = 8), Brazil (*n* = 10), Australia (*n* = 11), and Poland (*n* = 12). Only Netherlands (*n* = 3) achieved a lower score than the US, because there was only a response to one of four elements.

Detailed scores are reported in Supplementary Table S2.

When asked about the healthcare services most used in their country to collect information on digital health (*What kinds of sources and tools are mainly used in your country to collect information on digital health? Healthcare services*), respondents indicated primary care (mean = 4) as the most used on average, followed by pharmacies (mean = 3.6), emergency support information systems (mean = 3.5), drug monitoring systems (mean = 3.4), local health monitoring systems (mean = 3.34), smart hospitals and providers and mobile-health applications (mean = 3.3 each); the ones scoring the lowest were online payments for healthcare services (mean = 2.6), online bookings for healthcare services and Ambulance monitoring systems (mean = 3 each), electronic patient web portals (mean = 3.2).

India scored the highest (mean = 4.91), whereas Australia scored the lowest (mean = 2.36). This is reflected in the overall scores, where India continues leading (*n* = 54), followed by Hong Kong and South Korea (*n* = 42 each), then Canada and the US (*n* = 36 each). The countries performing the worst are Australia (*n* = 26), Italy (*n* = 29), Brazil (*n* = 30), Netherlands (*n* = 31), and Poland (*n* = 33).

**Figure 1:**
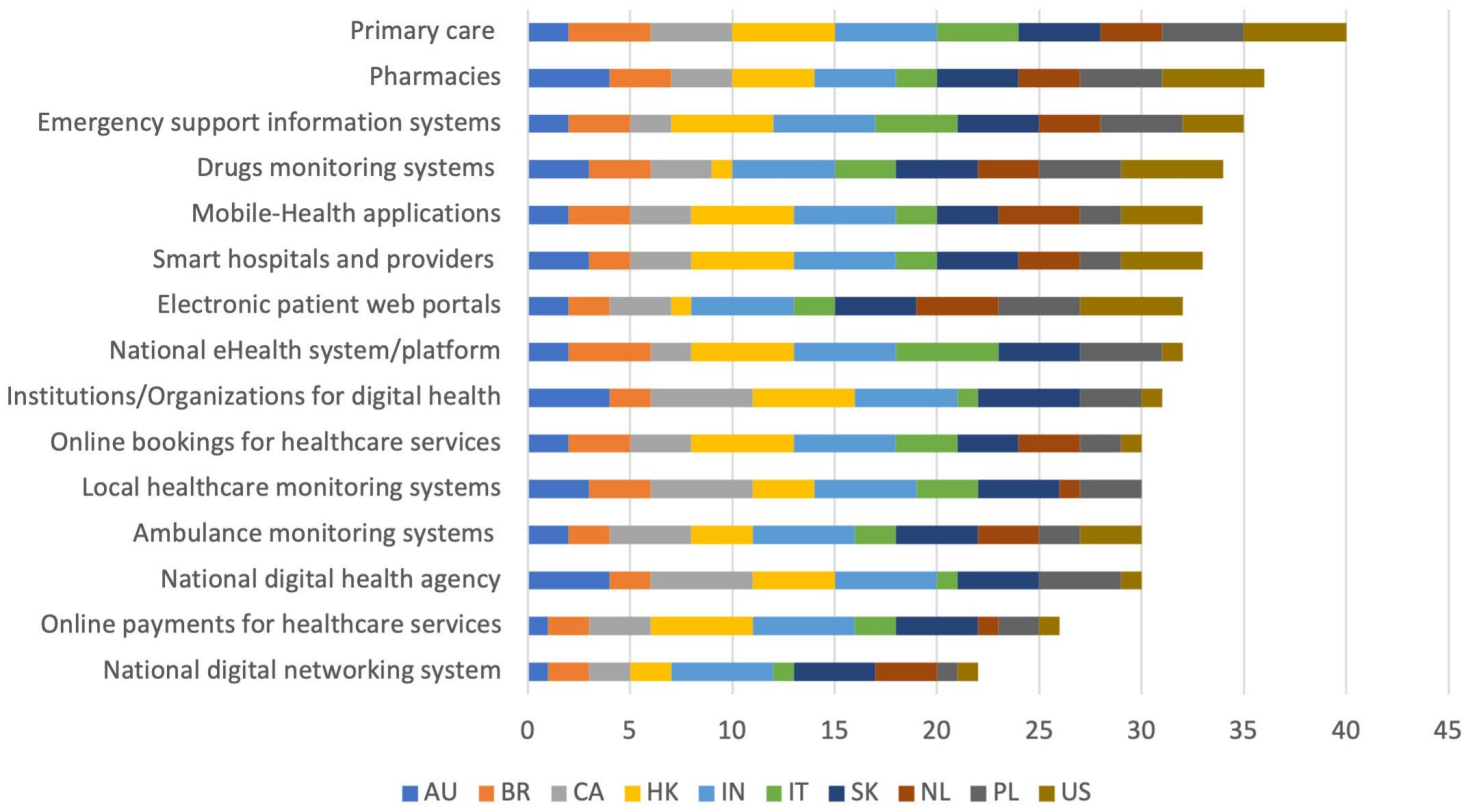
Cumulative scores on items from the question “What kinds of sources and tools are mainly used in your country to collect information on digital health? Centralized infrastructure and Healthcare services.”

Analyzing the cumulative scores achieved by the single items, it can be noted that primary care (*n* = 40), pharmacies (*n* = 36), and emergency support information systems (*n* = 35) achieve the highest scores. In contrast, national digital networking systems (*n* = 22), online payment for healthcare services (*n* = 26), and national digital health agencies (*n* = 30) performed the worst. The cumulative scores obtained by every single item are shown in Figure 1. Detailed scores are reported in Supplementary Tables S2, S3.

### Actions taken to consolidate digital health communication

When asked about the tools most used in their country to communicate and disseminate results of digital health services (*What are the tools your government/country uses for the communication/dissemination of results of digital health services?*), respondents indicated institutional websites (mean = 4.4) as the most used on average, followed by video interviews of institutional representatives (mean = 3.9), the media and social media (mean = 3.8 each); the tools scoring the lowest were newspaper articles (mean = 3.67) and scientific publications (mean = 3.78).

**Figure 2:**
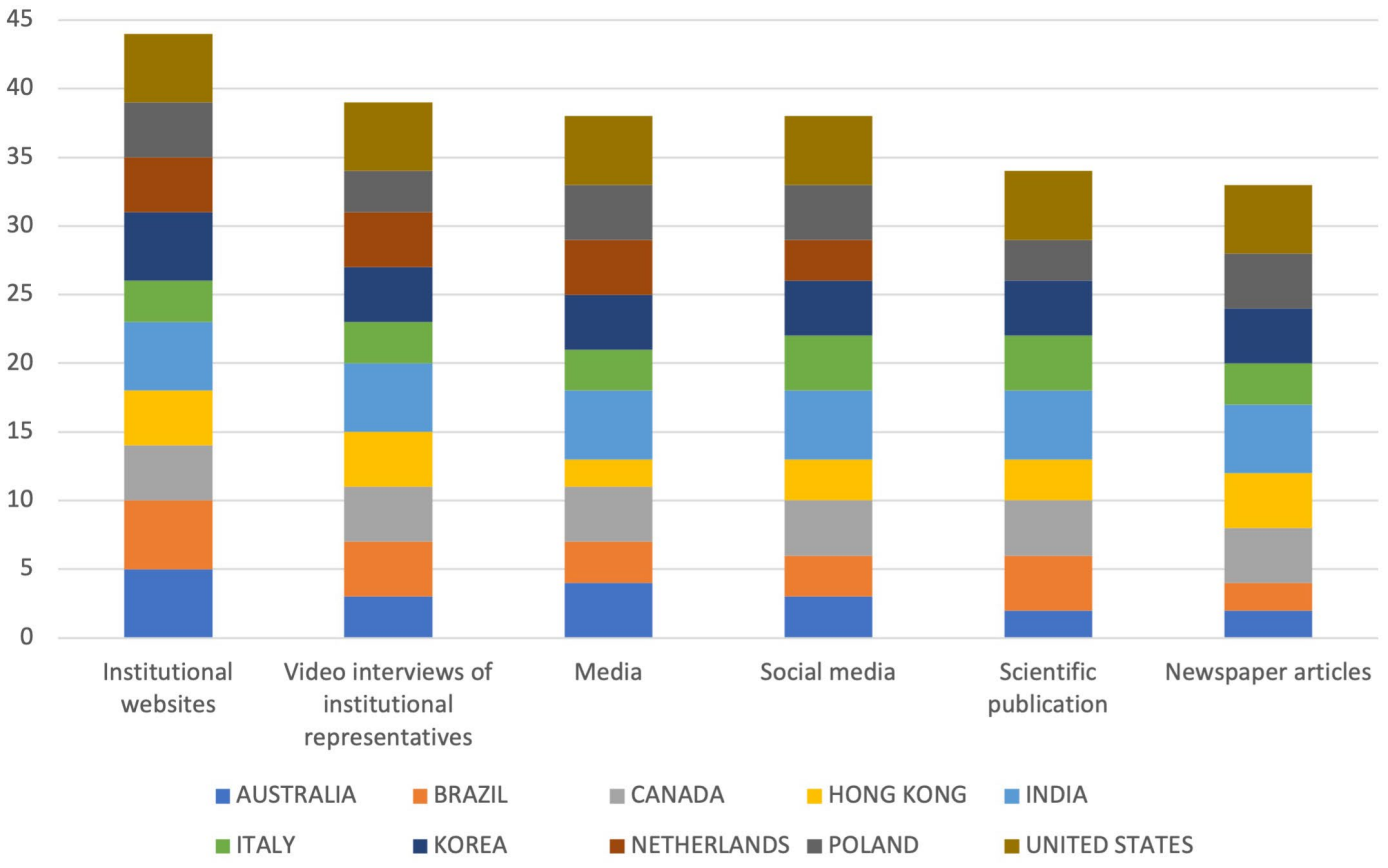
Cumulative scores on items from the question “What are the tools your Government/Country uses for the communication/dissemination of results of digital health services?”

The US and India scored the highest (mean = 5 each), whereas Australia scored the lowest (mean = 3.17). This is reflected in the overall scores, where the US and India keep leading (30 each), followed by South Korea (25), Hong Kong (24) and Brazil (21). The countries performing the worst are Australia (19), Hong Kong and Italy (20 each). Only Netherlands (15) performed worse than Australia, having responded to four out of six available items. Considering the cumulative scores, achieved by the single items, it can be noted that institutional websites (*n* = 44) and video interviews of institutional representatives (*n* = 39) achieve the highest scores, whereas newspaper articles (*n* = 33) and scientific publications (*n* = 34) performed the worst. The cumulative scores obtained by each single item is shown in Figure 2.

Detailed scores are reported in Supplementary Table S4.

**Figure 3:**
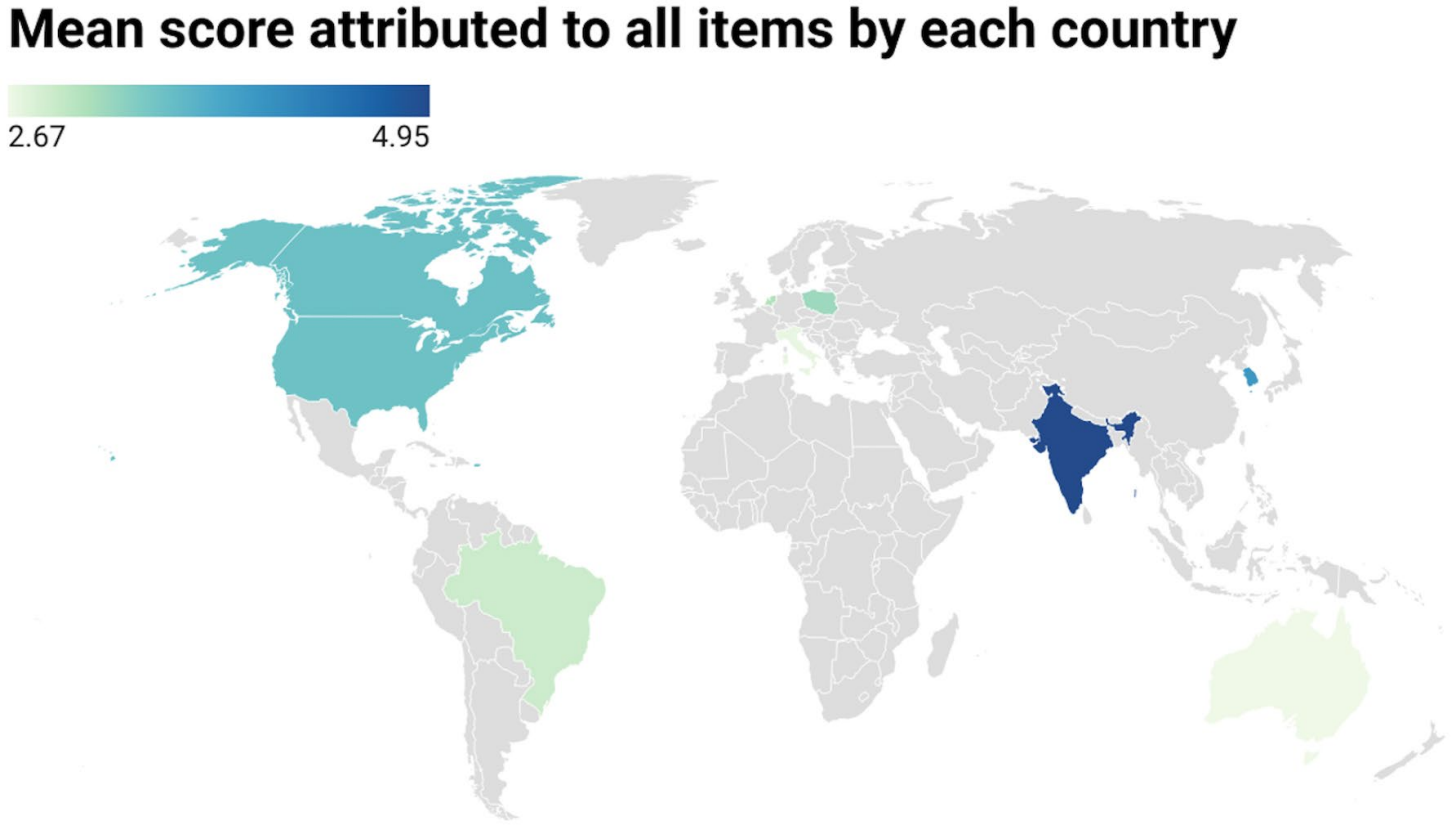
Mean score attributed to all items by each country.

Overall, India was the country with the highest mean score (mean = 4.95), followed by South Korea (mean = 4.00), Hong Kong (mean = 3.71), Canada (mean = 3.52), and the US (mean = 3.50). The countries scoring the lowest were Australia (mean = 2.67), Italy (mean = 2.67), Brazil (mean = 2.90), Netherlands (mean = 3.06), and Poland (mean = 3.19). A graphical representation on a world map can be seen in Figure 3.

**Figure 4:**
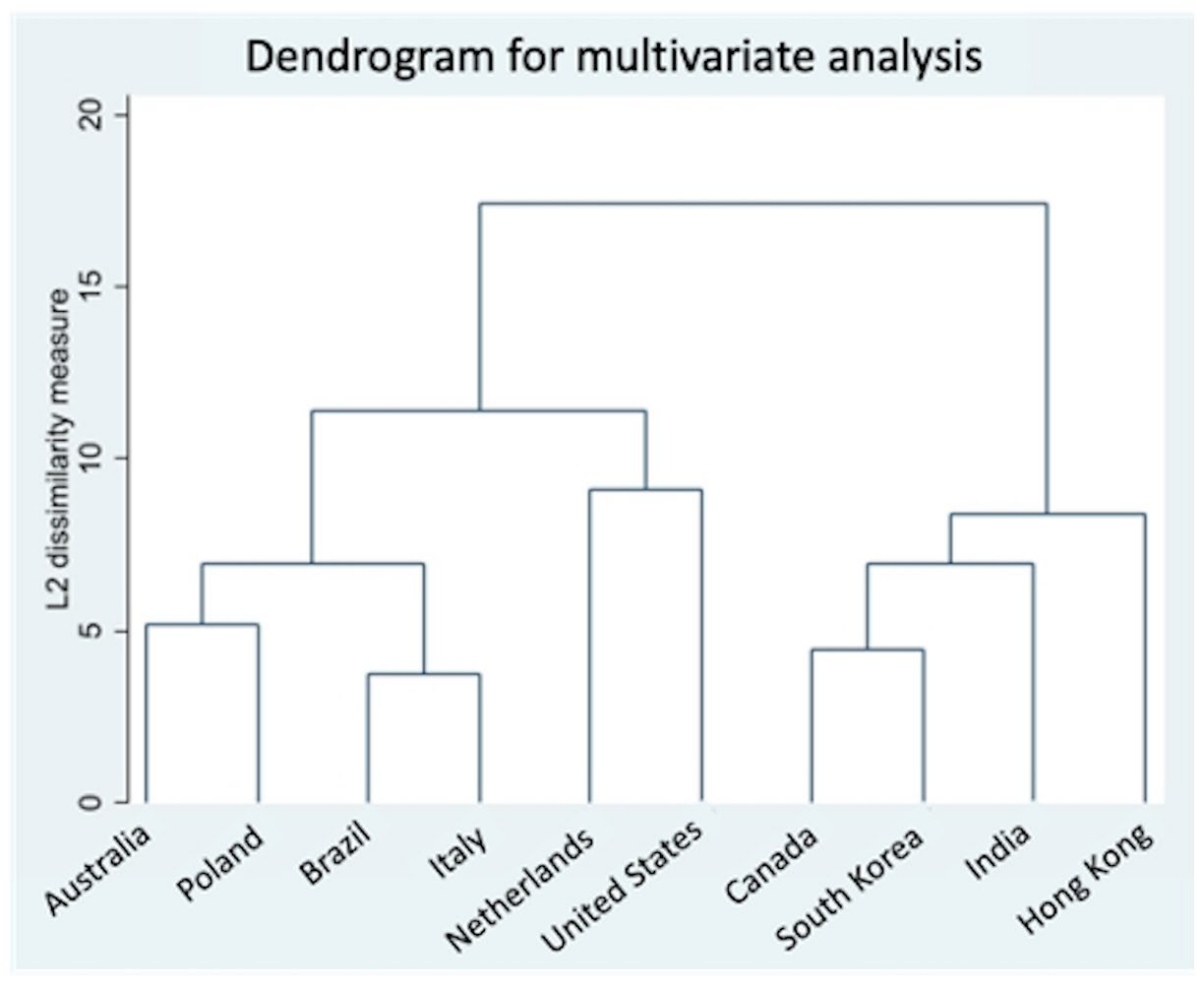
Dendrogram for multivariate analysis.

Finally, we performed a cross-country analysis on 220 survey outcomes (22 items in 10 countries) to sort the different countries according to similarities in how they responded to the questions. Two major clusters emerged, one containing six countries (Australia, Poland, Brazil, Italy, Netherlands, and the United States) and four countries (Canada, South Korea, India, and Hong Kong), respectively. The resulting dendrogram can be seen in Figure 4.

The overall Cronbach’s alpha showed a high concordance (scale reliability coefficient: 0.91; *p* < 0.05).

### Tools for communication/dissemination of results

**Figure 5:**
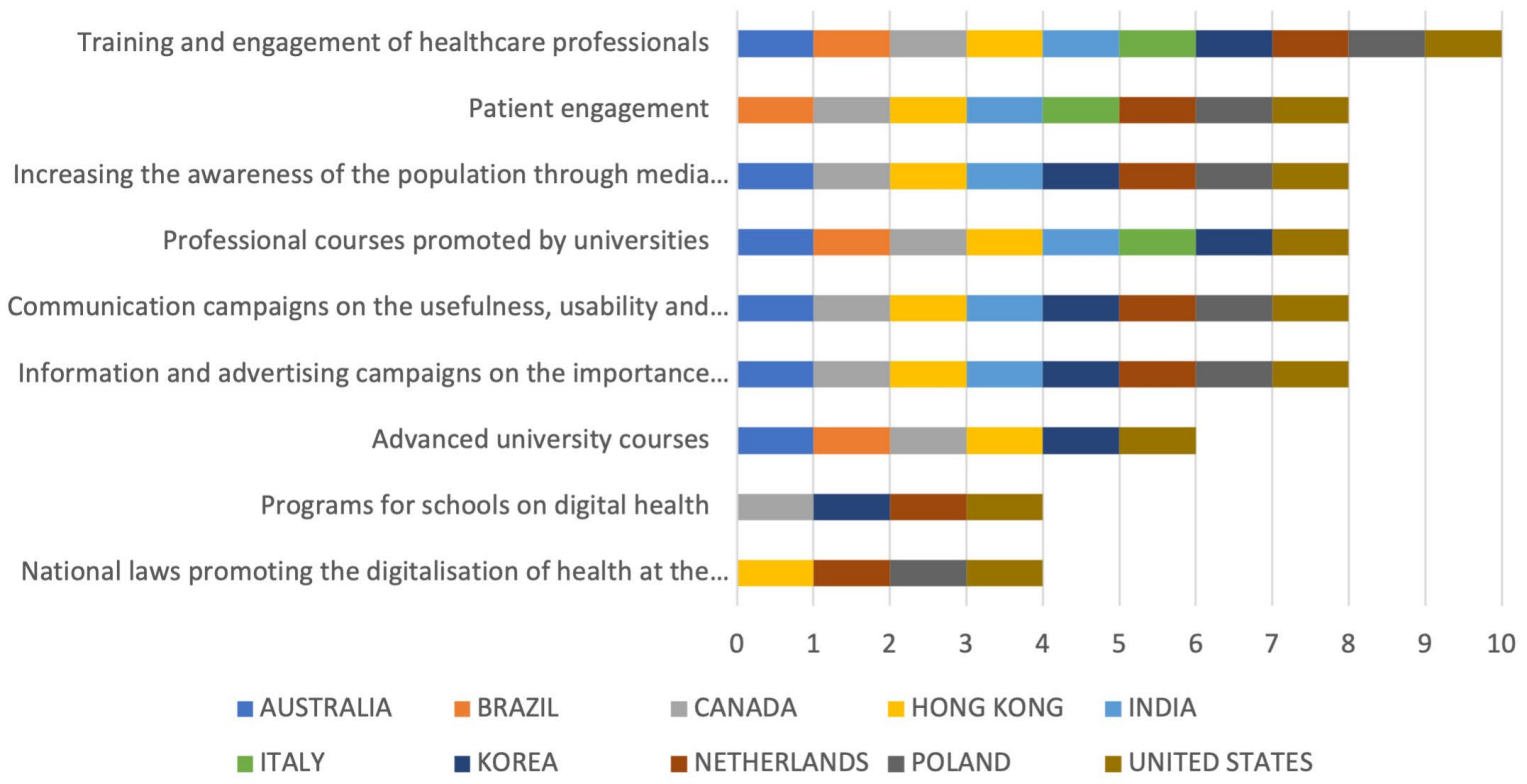
Cumulative scores on items for communication/dissemination of results.

Concerning the tools used for communication/dissemination of results (*What has been done at a national level to develop a consolidated communication of digital health technologies?*), the most adopted strategy was the training and engagement of healthcare professionals (*n* = 10), followed by information and advertising campaigns on the importance of digital health, communication campaigns on the usefulness, usability, and availability of digital health, professional courses promoted by universities, increasing the awareness of the population through media and press, and patient engagement (*n* = 8 each). The least adopted strategies were advanced university courses (*n* = 6) and programs for schools on digital health (*n* = 4). The countries having the most programs in place were the US and Canada (*n* = 8 each), followed by Hong Kong and South Korea (*n* = 7 each), India, Australia, and Netherlands (*n* = 6 each). The countries performing the worst were Italy (*n* = 3), Brazil (*n* = 4), and Poland (*n* = 5). The detailed results are shown in Figure 5.

### Barriers to implement digital health use

**Figure 6:**
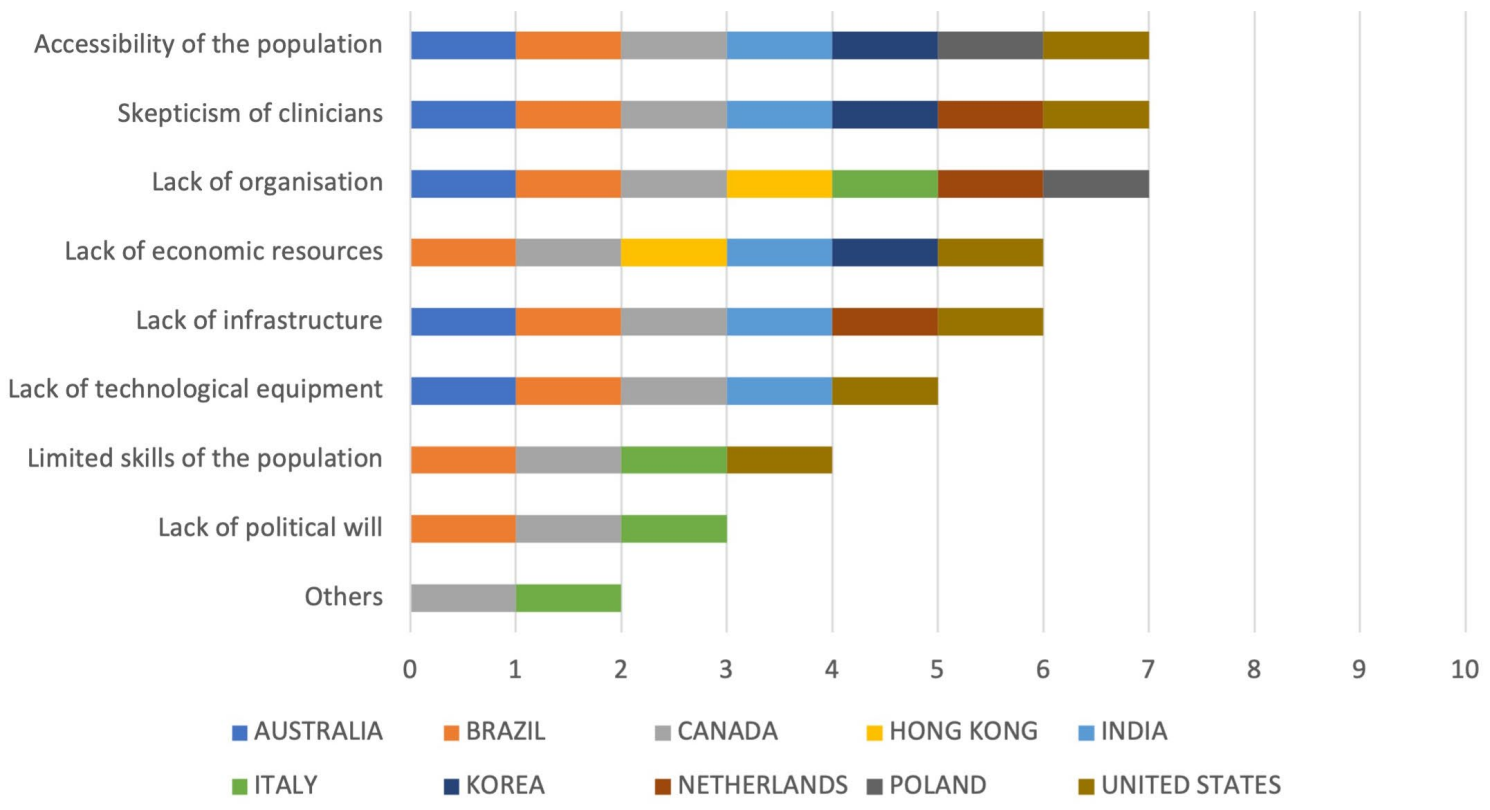
Cumulative scores on barriers to implement digital health use.

From this item, the barriers most perceived as an obstacle to implementing digital health (*Please indicate if, in your experience, any of the following are barriers to implementing digital health use*) were the lack of organization, the skepticism of clinicians, and the accessibility of the population (*n* = 7 each), followed by the lack of infrastructures and the lack of economic resources (*n* = 6 each). The least perceived barriers were the lack of political will (*n* = 3), the limited skills of the population (*n* = 4), and the lack of technological equipment (*n* = 5). The lack of financial incentives and the payment model for healthcare professionals were also indicated as barriers in the “Others” section. Figure 6 provides an overview of cumulative scores on barriers to implement digital health use, sorted by item.

Detailed scores are reported in Supplementary Table S5.

In the third section, an open-ended question addressed the priorities of intervention in digital health. Brazil and the United States referred to their national plans or strategy (National Digital Health Strategy and Federal Health IT Strategic Plan, respectively). The most endorsed priorities are the adoption of data-driven approaches and models (*n* = 6), telehealth (*n* = 5), data interoperability, healthcare workers & citizens, and clinical AI (each *n* = 4). Other priorities include the evaluation and uptake of innovation and biosurveillance (both *n* = 3), Electronic Health Records (EHRs) and population health (both *n* = 2).

The detailed results are reported in Table 2.

**Table 2:** Priorities indicated by national states representatives in open questions.

Topics	AU	BR	CA	HK	IN	IT	SK	NL	PL	US
Data interoperability	X				X	X	X			
Data-driven approach and model	X	X		X	X	X				X
Evaluation and uptake of innovation	X		X					X		
Telehealth		X	X	X		X		X		
Clinical AI		X		X		X			X	
EHRs					X		X			
Population health							X			X
Healthcare workers and citizens		X			X			X	X	
Biosurveillance					X	X		X		

AU, Australia; BR, Brazil; CA, Canada; HK, Hong Kong; IN, India; IT, Italy; SK, South Korea; NL, Netherlands; PL, Poland; US, United States.

## Discussion

GDHP is constantly investigating the evolution of healthcare digitalization, focusing on competencies in these areas and identifying factors relating to them (13). This survey section aimed to understand attitudes towards promotion and communication. Important differences among countries were already described in the literature, highlighting how self-rated promotion efforts might disregard national policies (19), thus the need to consult policymakers about their vision. Moreover, the heterogeneity characterizing the responding countries allows for hypothesizing what might possibly be the impact of the socio-economic condition of the operators on their work (20). This statement is backed by the dendrogram distribution of involved countries, which evidenced two main groups and variously distributed subgroups. Nevertheless, the interest in guaranteeing adequate communication in health keeps growing worldwide, based on the evidence that “informing, influencing, and motivating individual, institutional, and public audiences about important health issues” might steer the care process itself (21). Despite their differences, countries demonstrated a high internal consistency, which implies an adequate selection of survey items (22). Based on variance, the most heterogeneous group of answers was related to the question “What kind of sources and tools are mainly used in your country to collect information on digital health?,” highlighting how countries take care of data gathering differently.

Health data poverty is among the major obstacles to digital health evolution and, simultaneously, among the most effective tools for reporting and, thus, reducing inequities (23). Primary care and pharmacies were identified as the most frequently used settings for collecting data. This outcome has already been confirmed by literature, by reporting territorial facilities’ potential during the COVID-19 pandemic, through contact tracing and follow-up visits (24). The great literature production derived from gathered data is an example of how these settings helped support health systems evolution (25), with a recognized importance of collected information for the evolution of clinical knowledge and a step forward for the global knowledge ecosystem (26).

eHealth systems/platforms (mean score of 3.56) were identified as the best tools to collect information in digital health. Europe already recognized their potential to improve health by developing the European Health Data Space, which aims to support their evolution to make full use of the potential offered by a safe and secure exchange, use and reuse of health data (27, 28). Moreover, with the evolution of Big Data, the fields of precision medicine and public health will converge into precision public health, the study of biological and genetic factors supported by large amounts of population data (29). The use of big data gives the opportunity to for institutional bodies to draw on huge databases such as the European Union aims to do with the Digital Europe Program (30). Indeed, institutional websites (mean score of 4.4) were identified as the main means to communicate digital health results, whereas newspaper articles (3.67), and scientific publications (3.78) were those that work less.

The problem of health literacy, considered as the patients’ ability to face technical medicine topics, is controversial since technical language and sectorial knowledge might generate confusion and misunderstandings, slowing the care process (31). Patients’ engagement falls under the theme of personalized medicine, as well as among the main barriers highlighted through this GDHP survey (accessibility of the population). Previous studies have already showed how considering patients’ points of view and integrating social sciences and medical humanities to diagnose, define, design, and test communication interventions might optimize interventions’ effectiveness and impact (32, 33). Thus, there is a need for policymakers to take this theme into account.

Most countries identified the lack of organization as the biggest barrier to digital health use implementation. Vaughn et al. in 2019 carried out a systematic review on this theme and identified five main domains characterizing struggling healthcare organizations: poor organizational culture, inadequate infrastructure, lack of a cohesive mission, system shocks (i.e., events such as leadership turnover, new electronic health record system or organizational scandals that detract from daily operations), and dysfunctional external relations with other hospitals, stakeholders, or governing bodies (34). There is no simple solution to a problem deriving from multiple variables, although a systematization of coordination strategies and mechanisms might reduce its negative impact of it. A possible solution might be a supranational coordination of processes and systems, aiming at improving and achieving universal standards, as shared by the GDHP vision.

Another hot topic regarding obstacles to progress is the skepticism of clinicians towards digitalization. As reported by various sources, digital health implementation is a process whose development is rooted in bureaucracy, such as constraints in reimbursement and credentialing and administrative and training burdens (35, 36).

In addition to policy barriers, clinicians usually report three main areas of misbelief on the matter, namely error risk, lack of interaction and fear of becoming not necessary for the process of care (37). Finally, older adults as a group are on the negative side of the digital divide, which brings a mistrust towards new technologies of older doctors (38). The conservatism of the medical community might reduce the possibility of including mentoring in adapting young professionals to the use of eHealth solutions (37). Once again, the COVID-19 pandemic forced health professionals to adapt their work routine to the required situation, following learning through exposure strategy and breaking down generational barriers (37). The effectiveness of this shock therapy is supported by the collected open-ended answers to the third question, “priorities of intervention in digital health,” where a data-driven approach and telehealth were identified as the most endorsed priorities, both widely adopted during the pandemic, respectively for risk assessment and tele-visits. The opportunity to take advantage of an emergency situation, such as COVID-19, to support the change of health structures is one of the building pillars of Next Generation EU, the European project aiming to support the digital transition of health (39).

This paper aims to serve as a guide to policymakers involved in this project in terms of evidence on how to distribute resources. The main strength of this study is the novelty of its subject. There is currently no similar international forum to share best practices and enable co-working in digital health. Sharing this survey with political representatives guarantees the value of the received feedback. The publication of a whitepaper on the development of standard benefits and outcome measurements guided the researchers in following the footsteps of the previous GDHP literature (15). Finally, the heterogeneous sample derived from distinct geographical regions represents the topics worldwide.

There are limitations to our study as well. First, GDHP produces voluntary surveys, implying that there might be participation or non-response bias. Moreover, responders particularly interested to the proposed topic may have been more likely to send feedback to the survey than those who did not strongly feel the matter. This study had a low response rate of 34.5% (*n* = 10). Dividing the reached countries with a geographical method, the response of African countries was the lowest (0 out of 2 countries). The response of North America was the highest (2 out of 2 countries), followed by Oceania (1 out of 2 countries), Asia (3 out of 9 countries), Europe (3 out of 10 countries), and South America (1 out of 4 countries). The uneven representation of the geographical regions in the partnership does not allow us to statistically evaluate these numbers.

Finally, no significant differences were reported when we stratified answers by socio-cultural aspect (Western world, Eastern world) and by health care system model (Beveridge model, Bismarck model, National Health Insurance model, Out-Of-Pocket model). This may be due to the low response rate that does not allow statistical evaluations, but it may be also related to the fact that those countries and territories shared common agreements on digital health development and innovations (40).

This section of the survey included 5 questions and required a pragmatic study of national needs to be answered. The survey length may have discouraged responses, limiting the sample size to those with adequate time. This survey also lacked a scientifically validated method during its development due to the novelty of this topic. More studies are needed to confirm the current data. Finally, the low response number did not allow a generalization of results.

## Conclusion

This study highlights the major tools and instruments used among different GDHP Countries to the implementation of digital health innovations. The skepticism among clinicians and lack of accessibility for the general population have been detected as major barriers to the use of digital health technologies. For that reason, identifying strategies that would communicate the value of health care information technology to healthcare professionals and identify ways to increase accessibility of digital health technology to populations is a particular imperative. Lack of communication creates situations where medical errors can occur, undermining the foundations of the doctor-patient relationships. Medical errors, especially those caused by a failure to communicate, are a pervasive problem in today’s health care organizations. Effective communication programs for clinicians and the general population and an improved digital health literacy (both for clinicians and citizens) will be the key for the real implementation of future digital health technologies.

## Data availability statement

The original contributions presented in the study are included in the article/Supplementary material, further inquiries can be directed to the corresponding author.

## Author contributions

FC, AG, FAC, GA, AM, FB, CP, AL, and WR contributed to revise work for important intellectual content, gave the final approval of the version to be published, and agreed on all aspects of the work, especially concerning its accuracy and integrity. Further specific activities have been distributed as follows: FC conceived the research hypothesis. FC and WR designed the study. AG built the questionnaire for the survey. GA, FAC, and AM performed the data extraction. FB and CP shaped the manuscript with input from the entire team. All authors contributed to the article and approved the submitted version.

## Conflict of interest

The authors declare that the research was conducted in the absence of any commercial or financial relationships that could be construed as a potential conflict of interest.

## Publisher’s note

All claims expressed in this article are solely those of the authors and do not necessarily represent those of their affiliated organizations, or those of the publisher, the editors and the reviewers. Any product that may be evaluated in this article, or claim that may be made by its manufacturer, is not guaranteed or endorsed by the publisher.

## References

[1] Schwab Klaus. The fourth industrial revolution. World Economic Forum, editor. Available at: https://www.weforum.org/about/the-fourth-industrial-revolution-by-klaus-schwab (Accessed November 23, 2022).

[2] Frank SR. Digital health care--the convergence of health care and the internet. J Ambul Care Manage. (2000) 23:8–17. doi: 10.1097/00004479-200004000-00003 10848396

[3] Mathews SC McShea MJ Hanley CL Ravitz A Labrique AB Cohen AB. Digital health: a path to validation. NPJ Digit Med. 2:38. doi: 10.1038/s41746-019-0111-3 PMC6550273 31304384

[4] World Health Organization. (2021). Global strategy on digital health 2020-2025. Available at: https://apps.who.int/iris/handle/10665/344249 (Accessed November 23, 2022).

[5] Carini E Villani L Pezzullo AM Gentili A Barbara A Ricciardi W. The impact of digital patient portals on health outcomes, system efficiency, and patient attitudes: updated systematic literature review. J Med Internet Res. (2021) 23:1–20. doi: 10.2196/26189 PMC8459217 34494966

[6] Goldzweig CL Orshansky G Paige NM Towfigh AA Haggstrom DA Miake-Lye I. Electronic patient portals: evidence on health outcomes, satisfaction, efficiency, and attitudes: a systematic review. Ann Intern Med. (2013) 159:677–87. doi: 10.7326/0003-4819-159-10-201311190-00006, PMID: 24247673

[7] Han HR Gleason KT Sun CA Miller HN Kang SJ Chow S. Using patient portals to improve patient outcomes: systematic review. JMIR Hum Factors. (2019) 6:e15038. doi: 10.2196/15038 31855187 PMC6940868

[8] Gentili A Failla G Melnyk A Puleo V Di TGL Ricciardi W. The cost-effectiveness of digital health interventions: a systematic review of the literature. Front Public Health. (2022) 10:2656. doi: 10.3389/fpubh.2022.787135 PMC9403754 36033812

[9] Jiang X Ming W-K You JHS. The cost-effectiveness of digital health interventions on the management of cardiovascular diseases: systematic review. J Med Internet Res. (2019) 21:e13166. doi: 10.2196/13166 31210136 PMC6601257

[10] Cascini F Santaroni F Lanzetti R Failla G Gentili A Ricciardi W. Developing a data-driven approach in order to improve the safety and quality of patient care. Front Public Health. (2021) 9:667819. doi: 10.3389/fpubh.2021.667819, PMID: 34095071 PMC8175645

[11] Global Digital Health Partnership – Summit. (2018). In Washington DC. Available at: https://www.google.com/search?q=Global+Digital+Health+Partnership+–+24+-+25+April+2018+summit+Washington+DC%2C+United+States+of+America+communique+(2018&oq=Global+Digital+Health+Partnership+−+24+−+25+April+2018+summit+Washington+DC%2C+United+States+of+America+communique+(2018&aqs=chrome.69i57j69i64.434j0j4&sourceid=chrome&ie=UTF-8 (Accessed November 23, 2022).

[12] Global Digital Health Partnership. Available at: https://gdhp.health/ (Accessed May 10, 2023).

[13] The Global Digital Health Partnership. Available at: https://www.healthit.gov/topic/global-digital-health-partnership (Accessed November 23, 2022).

[14] Makeham M Donohoe T. (2019). Measuring benefits. Global Digital Health Partnership. Available at: https://www.healthit.gov/topic/global-digital-health-partnership (Accessed May 10, 2023).

[15] Loo Gee B Makeham M Ecclestone R Lubbers C. (2020). Benefits realisation: sharing insights. Sidney. Available at: https://www.healthit.gov/sites/default/files/page/2021-01/GDHP-BenefitsRealisation Sharing Insights.pdf (Accessed November 23, 2022).

[16] Omboni S Padwal RS Alessa T Benczúr B Green BB Hubbard I. The worldwide impact of telemedicine during COVID-19: current evidence and recommendations for the future. Connect Health. (2022) 1:7–35. doi: 10.20517/ch.2021.03 35233563 PMC7612439

[17] Wang Q Su M Zhang M Li R. Integrating digital technologies and public health to fight Covid-19 pandemic: key technologies, applications, challenges and outlook of digital healthcare. Int J Environ Res Public Health. (2021) 18:6053. doi: 10.3390/ijerph18116053 34199831 PMC8200070

[18] Donk M Theeuwes J. Prioritizing selection of new elements: bottom-up versus top-down control. Percept Psychophys. (2003) 65:1231–42. doi: 10.3758/bf03194848 14710958

[19] Tabak RG Reis RS Wilson P Brownson RC. Dissemination of health-related research among scientists in three countries: access to resources and current practices. Biomed Res Int. (2015) 2015:1–9. doi: 10.1155/2015/179156, PMID: 26495287 PMC4606183

[20] Kourtellos A. Modeling parameter heterogeneity in cross-country regression models. Front Econ Glob. (2011) 11:367–87. doi: 10.1108/S1574-8715(2011)0000011018

[21] Bernhardt JM. Communication at the core of effective public health. Am J Public Health. (2011) 94:2051–3. doi: 10.2105/ajph.94.12.2051 PMC1448586 15569948

[22] Shelby LB. Beyond Cronbach’s alpha: considering confirmatory factor analysis and segmentation. Hum Dimens Wildl. (2011) 16:142–8. doi: 10.1080/10871209.2011.537302

[23] Ibrahim H Liu X Zariffa N Morris AD Denniston AK. Health data poverty: an assailable barrier to equitable digital health care. Lancet Digit Health. (2021) 3:e260–5. doi: 10.1016/S2589-7500(20)30317-4 33678589

[24] Girum T Lentiro K Geremew M Migora B Shewamare S. Global strategies and effectiveness for COVID-19 prevention through contact tracing, screening, quarantine, and isolation: a systematic review. Trop Med Health. (2020) 48:91. doi: 10.1186/s41182-020-00285-w, PMID: 33292755 PMC7680824

[25] Ioannidis JPA Salholz-Hillel M Boyack KW Baas J. The rapid, massive growth of COVID-19 authors in the scientific literature. R Soc Open Sci. (2021) 8:210389. doi: 10.1098/rsos.210389, PMID: 34527271 PMC8422596

[26] Verspoor K. The evolution of clinical knowledge during COVID-19: towards a global learning health system. Yearb Med Inform. (2021) 30:176–84. doi: 10.1055/s-0041-1726503 34479389 PMC8416229

[27] de Keersmaecker S. A European Health Data Space for people and science [Internet]. European Commission Press Release (2022). Available from: https://ec.europa.eu/commission/presscorner/detail/en/ip_22_2711 (Accessed May 26, 2023).

[28] Horgan D Hajduch M Vrana M Soderberg J Hughes N Omar MI. European health data – an opportunity now to grasp the future of data-driven healthcare. Healthcare. (2022) 10:1629. doi: 10.3390/healthcare10091629 36141241 PMC9498352

[29] Velmovitsky PE Bevilacqua T Alencar P Cowan D Morita PP. Convergence of precision medicine and public health into precision public health: toward a big data perspective. Front Public Health. (2021) 9:305. doi: 10.3389/fpubh.2021.561873 PMC8055845 33889555

[30] European Commission. (2022). The digital Europe Programme. Available at: https://digital-strategy.ec.europa.eu/en/activities/digital-programme

[31] Vollbrecht H Arora V Otero S Carey K Meltzer D Press VG. Evaluating the need to address digital literacy among hospitalized patients: cross-sectional observational study. J Med Internet Res. (2020) 22:e17519. doi: 10.2196/17519 32496196 PMC7303835

[32] Würz A Nurm ÜK Ekdahl K. Enhancing the role of health communication in the prevention of infectious diseases. J Health Commun. (2013) 18:1566–71. doi: 10.1080/10810730.2013.840698 24298888

[33] Cascini F Beccia F Causio FA Muscat NA Ricciardi W. Editorial: digitalization for precision healthcare. Front Public Health. (2022) 10:1078610. doi: 10.3389/fpubh.2022.1078610 36530708 PMC9755876

[34] Vaughn VM Saint S Krein SL Forman JH Meddings J Ameling J. Characteristics of healthcare organisations struggling to improve quality: results from a systematic review of qualitative studies. BMJ Qual Saf. (2019) 28:74–84. doi: 10.1136/bmjqs-2017-007573 PMC6373545 30045864

[35] Cowan KE McKean AJ Gentry MT Hilty DM. Barriers to use of Telepsychiatry: clinicians as gatekeepers. Mayo Clin Proc. (2019) 94:2510–23. doi: 10.1016/j.mayocp.2019.04.018, PMID: 31806104

[36] Perry K Gold S Shearer EM. Identifying and addressing mental health providers’ perceived barriers to clinical video telehealth utilization. J Clin Psychol. (2020) 76:1125–34. doi: 10.1002/jclp.22770 30859573

[37] Sherrill AM Wiese CW Abdullah S Arriaga RI. Overcoming clinician technophobia: what we learned from our mass exposure to telehealth during the COVID-19 pandemic. J Technol Behav Sci. (2022) 7:547–53. doi: 10.1007/s41347-022-00273-3 36034538 PMC9391067

[38] McDonough CC. The effect of ageism on the digital divide among older adults. Gerontol Geriatr Med. (2016) 2:1–7. doi: 10.24966/GGM-8662/100008

[39] Alcidi C Gros D. Next generation EU: a large common response to the COVID-19 crisis. Inter Econ. (2020) 55:202–3. doi: 10.1007/s10272-020-0900-6 32834093 PMC7385205

[40] Cascini F Altamura G Failla G Gentili A Puleo V Melnyk A. Approaches to priority identification in digital health in ten countries of the Global Digital Health Partnership. Front Digit Heal. (2022) 4:4. doi: 10.3389/fdgth.2022.968953 PMC9632991 36339514

