# Supplementary material for: Strengthening and promoting digital health practice: results from a Global Digital Health Partnership’s survey
Source: Front Public Health. 2023 Jun 19;11:1147210. doi: 10.3389/fpubh.2023.1147210 (PMC10315462; doi:10.3389/fpubh.2023.1147210)
Supplement: Supplementary file 1 [file Table_1.pdf]

GDHP Supplementary Materials

Supplementary Table S1: list of the participating Countries, Organizations, and Territories composing GDHP

GDHP Participant Country	Name and title	Organization
Australia	Amy Winter<br>Senior Policy Advisor<br><br>Rick Sondalini<br>Senior Policy Advisor	Australian Digital Health Agency
Brazil	Dr Márcia Elizabeth Marinho da Silva<br>General Coordination of Innovation in Digital Systems<br><br>Thaís Lucena de Oliveira<br>General Coordination of Innovation in Digital Systems	Ministry of Health, Department of Informatics (DATASUS)
Canada	Michael Green<br>President and CEO<br><br>Simon Hagens<br>Senior Director, Performance Analytics	Canada Health Infoway

Hong kong	Dr Ngai Tseung Cheung<br>Head IT&Health Informatics<br><br>Dr Joan Hung<br>Principal Assistant Secretary	Electronic Health Record Office, Food & Health Bureau, Government of Hong Kong
Italy	Dr Fidelia Cascini<br>Università catt	Italian Ministry of Health
India	Lav Jaiswal<br>Director (eHealth)<br><br>Lav Agarwal<br>Joint Secretary	Ministry of Health and Family Welfare, Government of India
Netherlands	Herko Coomans<br>International Digital Health Coordinator<br><br>Roger Lim<br>Senior Policy Officer	Ministry of Health, Welfare and Sport, The Netherlands
Poland	Hubert Życiński<br>Head of Unit	Ministry of Health<br>(Department of Innovation)
South Korea	Dr Hun-Sung Kim<br>Associate Professor	The Catholic University of Korea
United States of America	Dr Aisha Hasan<br>Head of Global Health IT	U.S. Department of Health and Human Services

Supplementary Table S2. What kinds of sources and tools are mainly used in your country to collect information on digital health? Questions on centralized infrastructure. (AU: Australia; BR: Brazil; CA: Canada; HK: Hong Kong; IN: India; IT: Italy; SK: South Korea; NL: Netherlands; PL: Poland; US: United States; OA: Overall).

	AU	BR	CA	HK	IN	IT	SK	NL	PL	US	OA	Mean
National eHealth system/platform	2	4	2	5	5	5	4	-	4	1	32	3.56
National digital health agency	4	2	5	4	5	1	4	-	4	1	30	3.33
Institutions/Organizations for digital health	4	2	5	5	5	1	5	-	3	1	31	3.44
National digital networking system	1	2	2	2	5	1	4	3	1	1	22	2.2
AVERAGE	2.75	2.5	3.5	4	5	2	4.25	3	3	1	28.75	

Supplementary Table S3. What kinds of sources and tools are mainly used in your country to collect information on digital health? Questions on healthcare services. (AU: Australia; BR: Brazil; CA: Canada; HK: Hong Kong; IN: India; IT: Italy; SK: South Korea; NL: Netherlands; PL: Poland; US: United States; OA: Overall).

	AU	BR	CA	HK	IN	IT	SK	NL	PL	US	OA	Mean
Emergency support information systems	2	3	2	5	5	4	4	3	4	3	35	3.5
Ambulance monitoring systems	2	2	4	3	5	2	4	3	2	3	30	3
Smart hospitals and providers	3	2	3	5	5	2	4	3	2	4	33	3.3
Local healthcare monitoring systems	3	3	5	3	5	3	4	1	3	-	30	3.3
Online bookings for healthcare services	2	3	3	5	5	3	3	3	2	1	30	3
Online payments for healthcare services	1	2	3	5	5	2	4	1	2	1	26	2.6

Primary care	2	4	4	5	5	4	4	3	4	5	40	4
Electronic patient web portals	2	2	3	1	5	2	4	4	4	5	32	3.2
Mobile-Health applications	2	3	3	5	5	2	3	4	2	4	33	3.3
Pharmacies	4	3	3	4	4	2	4	3	4	5	36	3.6
Drugs monitoring systems	3	3	3	1	5	3	4	3	4	5	34	3.4
AVERAGE	2.36	2.73	3.27	3.82	4.91	2.64	3.82	2.82	3	3.6	32.64	

Supplementary Table S4. What are the tools your government/country uses for the communication/dissemination of results of digital health services? (AU: Australia; BR: Brazil; CA: Canada; HK: Hong Kong; IN: India; IT: Italy; SK: South Korea; NL: Netherlands; PL: Poland; US: United States; OA: Overall).

	AU	BR	CA	HK	IN	IT	SK	NL	PL	US	OA	Mean
Media	4	3	4	2	5	3	4	4	4	5	38	3.8
Scientific publication	2	4	4	3	5	4	4		3	5	34	3.8
Institutional websites	5	5	4	4	5	3	5	4	4	5	44	4.4
Newspaper articles	2	2	4	4	5	3	4		4	5	33	3.7
Social media	3	3	4	3	5	4	4	3	4	5	38	3.8
Video interviews of institutional representatives	3	4	4	4	5	3	4	4	3	5	39	3.9
AVERAGE	2.80	3.13	3.48	3.45	4.92	2.74	3.99	3.44	3.54	4.74	35.79	

Supplementary Table S5. Please indicate if, in your experience, any of the following are barriers to implementing digital health use. (AU: Australia; BR: Brazil; CA: Canada; HK: Hong Kong; IN: India; IT: Italy; SK: South Korea; NL: Netherlands; PL: Poland; US: United States; OA: Overall, AVG: Average)

	AU	BR	CA	HK	IN	IT	SK	HL	PL	US	YES	NO
Lack of infrastructure	✓	✓	✓	X	✓	X	X	✓	X	✓	6	4
Lack of technological equipment	✓	✓	✓	X	✓	X	X	X	X	✓	5	5
Lack of political will	X	✓	✓	X	X	✓	X	X	X	X	3	7
Lack of economic resources	X	✓	✓	✓	✓	X	✓	X	X	✓	6	4
Lack of organisation	✓	✓	✓	✓	X	✓	X	✓	✓	X	7	3
Skepticism of clinicians	✓	✓	✓	X	✓	X	✓	✓	X	✓	7	3
Limited skills of the population	X	✓	✓	X	X	✓	X	X	X	✓	4	6
Accessibility of the population	✓	✓	✓	X	✓	X	✓	X	✓	✓	7	3
Others	X	X	✓	X	X	✓	X	X	X	X		
YES	5	8	9	2	5	4	3	3	2	6		
NO	4	1	0	7	4	5	6	6	7	3		
